# Genetic resources of narrow-leaved lupine (Lupinus angustifolius L.) and their role in its domestication and breeding

**DOI:** 10.18699/VJ21.070

**Published:** 2021-10

**Authors:** M.A. Vishnyakova, E.V. Vlasova, G.P. Egorova

**Affiliations:** Federal Research Center the N.I. Vavilov All-Russian Institute of Plant Genetic Resources (VIR), St. Petersburg, Russia; Federal Horticultural Research Center for Breeding, Agrotechnology and Nursery, Moscow, Russia; Federal Research Center the N.I. Vavilov All-Russian Institute of Plant Genetic Resources (VIR), St. Petersburg, Russia

**Keywords:** narrow-leaved lupine ; ., genetic resources, ex situ collections, diversity, gene pool, intraspecif ic differentiation, wild forms, люпин узколистный, генетические ресурсы, коллекции ex situ, разнообразие, генофонд, внутривидовая дифференциация, дикие формы

## Abstract

Narrow-leaved lupine (Lupinus angustifolius L.) is a cultivated multipurpose species with a very short history of domestication. It is used as a green manure, and for feed and food. This crop shows good prospects for use in pharmacology and as a source of f ish feeds in aquaculture. However, its genetic potential for the development of productive and adaptable cultivars is far from being realized. For crop species, the genetic base of the cultivated gene pool has repeatedly been shown as being much narrower than that of the wild gene pool. Therefore, eff icient utilization of a species’ genetic resources is important for the crop’s further improvement. Analyzing the information on the germplasm collections preserved in national gene banks can help perceive the worldwide diversity of L. angustifolius genetic resources and understand how they are studied and used. In this context, the data on the narrow-leaved lupine collection held by VIR are presented: its size and composition, the breeding status of accessions, methods of studying and disclosing intraspecif ic differentiation, the classif ications used, and the comparison of this information with available data on other collections. It appeared that VIR’s collection of narrow-leaved lupine, ranking as the world’s second largest, differed signif icantly from others by the prevalence of advanced cultivars and breeding material in it, while wild accessions prevailed in most collections. The importance of the wild gene pool for the narrow-leaved lupine breeding in Australia, the world leader in lupine production, is highlighted. The need to get an insight into the species’ ecogeographic diversity in order to develop cultivars adaptable to certain cultivation conditions is shown. The data on the testing of VIR’s collection for main crop characters valuable for breeders are presented. Special attention is paid to the study of accessions with limited branching as a promising gene pool for cultivation in relatively northern regions of Russia. They demonstrate lower but more stable productivity, and suitability for cultivation in planting patterns, which has a number of agronomic advantages. Analyzing the work with narrow-leaved lupine genetic resources in different national gene banks over the world helps shape the prospects of further activities with VIR’s collection as the only source of promising material for domestic breeding.

## Abstract (ru)

Люпин узколистный (Lupinus angustifolius L.) – окультуренный вид многоцелевого назначения с очень короткой историей доместикации. Его используют как сидеральную, кормовую, продовольственную культуру, в качестве корма в рыбоводстве и в фармакологии. Однако генетический потенциал вида для создания продуктивных и адаптивных сортов далеко не реализован. Неоднократно показана узкая генетическая основа окультуренного генофонда по сравнению с диким. Поэтому эффективное использование генетических ресурсов вида имеет важное значение для дальнейшего развития культуры. Разнообразие генетических ресурсов люпина узколистного в мире, степень их изученности и пути применения можно представить посредством анализа сведений о коллекциях гермоплазмы вида, сохраняемых в национальных генбанках разных стран. В контексте этого анализа в статье приведены сведения о коллекции люпина узколистного ВИР: ее ленности, составе, селекционном статусе образцов, методах изучения и выявления внутривидовой диффе- ренциации, используемых классификациях. Показано, что коллекция люпина узколистного ВИР, занимающая второе место в мире по числу образцов, значительно отличается от других преобладанием в ней сортов на- учной селекции и селекционного материала, в то время как в большинстве коллекций превалируют дикие формы. Освещено значение дикого генофонда в селекции люпина узколистного в Австралии – мировом лиде- ре производства культуры. Показана необходимость выявления эколого-географического разнообразия вида для создания сортов с адаптивными свойствами, соответствующими определенным условиям возделывания. Приведены данные оценки образцов коллекции ВИР по основным селекционно значимым признакам. Особое внимание уделено изучению образцов с ограниченным ветвлением, как перспективному генофонду для воз- делывания в сравнительно северных районах нашей страны. Они обладают меньшей, но более стабильной продуктивностью, пригодны для возделывания в загущенном посеве, что имеет целый ряд агротехнических преимуществ. Анализ работы с генетическими ресурсами люпина узколистного в различных национальных коллекциях мира способствует определению путей дальнейшей работы с коллекцией ВИР как единственным источником исходного материала для отечественной селекции.

## Introduction

Collections of plant genetic resources (PGR) are germplasm repositories maintained in many countries of the world. They preserve the global diversity of cultivated plants and their wild relatives, and differ from each other in age, number of preserved accessions, taxonomic diversity, mission, and purpose. Using these criteria to compare different PGR collections is not an easy task due to the absence of an integrated documentary source containing data on all the world’s plant germplasm holdings.

The genus Lupinus L. has a wide range of distribution. Species of the Old World (subgen. Lupinus L.) are widespread in the Mediterranean region and North Africa, while the New World lupines (subgen. Platycarpos (Wats.) Kurl.) occur in both Americas over a fairly wide range of latitudes and altitudes. Among the rich specific diversity of the genus, only a few species have been domesticated and widely introduced into agricultural production

The most comprehensive information on lupine collections, albeit far from complete, is the European Central Lupinus Database (DB). It contains data of 13,964 Lupinus L. accessions held in 13 genebanks of 10 countries.

Regrettably, the said European DB does not contain information about the collections of VIR and Belarus; moreover, the data presented there were last updated almost ten years ago. Other sources of information on the specific composition of the world’s lupine germplasm collections operate with either the same data (Święcicki et al., 2015) or even older figures (Buirchell, Cowling, 1998), or present only the total number of accessions for different species of the genus (Berger et al., 2013).

Currently, narrow-leaved lupine (L. angustifolius L.), also known as blue lupine, is the world’s leader in the area of cultivation among other cultivated Lupinus spp. It is the earliest and most plastic cultivated species and the only one adapted to relatively northern latitudes. Uses of this crop are very diverse. Traditionally, this is a green manure and forage crop. Of late, it has been intensively studied and used as a food crop. Quinolizidine alkaloids in seeds of different Lupinus spp. are of interest for pharmacology (Vishnyakova et al., 2020). Processed grains of various lupines, including the narrow-leaved one, have been used for several decades in fish farming to prepare feeds. This aspect of lupine utilization is promoted by numerous publications and web resources. One of the examples is the summary by B.D. Glencross (2001).

Nutritive value of narrow-leaved lupine is determined by its high content of protein (30–40 %), carbohydrates (40 %), oil (6 %), numerous minerals, vitamins, and other health-friendly ingredients. It is widely cultivated in Northern and Eastern Europe (Germany, the Netherlands, Poland, etc.), the United States, New Zealand, and Belarus. The world’s leader in the production and export of this crop, research into its genetic diversity, and most significant breeding achievements is Australia (Cowling, 2020; Vishnyakova et al., 2020). As far as the Russian Federation is concerned, the area of narrow-leaved lupine cultivation in 2019 reached 78,971 hectares, making this country one of the leading producers of this crop in the world http://www.fao.org/faostat/en/#data/QC

There are 27 cultivars listed in the State Register for Selection Achievements Admitted for Usage in Russia. Only seven of them were released in the past five years. This is not much on a national scale, considering the size of the country, but all of these cultivars were developed by Russian breeders. Intensification of plant breeding efforts obviously requires well-characterized source material. The only holder of such material in Russia is the global PGR collection of VIR.

The purpose of this publication is to analyze the worldwide diversity of narrow-leaved lupine GR preserved in national ex situ collections of different countries, with an emphasis on VIR’s collection, and discuss the prospects for effective utilization of these resources.

**When and where was L. angustifolius domesticated?**

Lupinus angustifolius L. is a very polymorphic species with high adaptive potential. The geographic range of narrowleaved lupine cultivation stretches from 30° S up to 60° N. Narrow-leaved lupine plants can tolerate temperatures as low as –9 °C (Kuptsov, Takunov, 2006). The known maximum altitude where this species occurs is 1,800 m above sea level. The soil pH gradient is 4.2–9.0. Annual precipitation in the areas where representatives of this species naturally occur is 200 to 1500 mm (Buirchell, Cowling, 1998). Narrow-leaved lupine plants are capable of growing on soils deficient in nitrogen and phosphorus. The diversity of morphological characters and adaptive properties of lupine has been triggered by an extensive environmental diversity of its habitats.

The center of origin for narrow-leaved lupine is the Mediterranean region. In the wild, L. angustifolius occurs much more frequently than other lupine species of the Old World and is still distributed throughout the Mediterranean (Cowling, 1986) as well as in Asia Minor, Transcaucasia, and Iran (Gladstones et al., 1998). Recent studies have shown that the greatest genetic diversity is found among the wild forms of narrow-leaved lupine in the western Mediterranean. There is a suggestion that it was the gene pool of the Iberian Peninsula that migrated eastwards, thus initiating the domestication of the species (Mousavi-Derazmahalleh et al., 2018a, b).

The process of domestication for narrow-leaved lupine presumably started in the 1930‒1940s, when the discovered alkaloid-free mutants (Sengbusch, 1931) enabled plant breeders in Germany and Sweden to develop the first forage cultivars (Maysuryan, Atabekova, 1974). Australian scientists, however, date it back to the 1960s and 1970s (Gladstones, 1970). At that time, plant breeders in Australia launched the development of cultivars combining in their genotype the maximum number of genes that determined the domestication syndrome, namely those controlling the absence of alkaloids (iuc), nonshattering of pods (le and ta), early flowering (Jul and Ku), permeability of the seed coat (moll), and white color of flowers and seeds (leuc) (Cowling, 2020). Breeding achievements in Australia led to a two to three times increase in the crop’s yield in the early 21st century across the main lupine cultivation areas in Western Australia since the release of the first cultivar in 1967 (French, Buirchell, 2005).

**History of the narrow-leaved lupine collection at VIR**

Since N.I. Vavilov’s times, VIR has been accumulating in its collection representatives of the cultivated flora together with accompanying wild relatives: species and varieties with certain individual properties that may be required and can be used by domestic plant breeders (Vavilov, 1925).

**Fig. 1.**
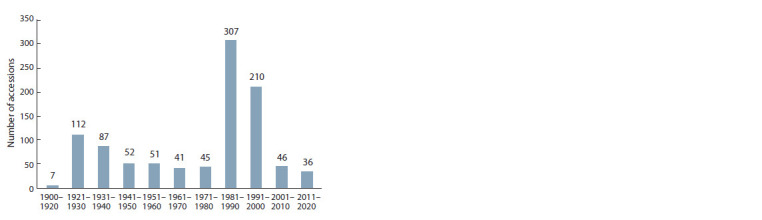
Historical dynamics of adding narrow-leaved lupine accessions to the collection.

The first accessions of narrow-leaved lupine were donated to VIR in 1919 (Fig. 1) by Prof. D.N. Pryanishnikov from Moscow Agricultural Institute (now Russian State Agrarian University – Timiryazev Moscow Agricultural Academy). The lupine collection, like most of the Institute’s collections, started to grow much more intensively with the coming of Nikolai Vavilov to the Institute. In the 1920s, N.I. Vavilov and the staff of VIR were busy ordering large-scale shipments of plant germplasm from various botanical gardens of France, England, Sweden, Poland, Czechoslovakia, Switzerland, Denmark, etc. Breeding material was actively procured from France (Vilmorin), England (Suttons Seeds & Bulbs) and Germany (Haage und Schmidt). Of crucial importance were Vavilov’s collecting missions to the centers of lupine origin. Valuable samples of L. angustifolius were collected in 1926–1927 in the Mediterranean countries: Italy, Greece, Spain, Algeria, and Palestine. For example, the accessions of Palestinian origin were characterized by earliness, rapid growth in the first half of the growing season, high yield of green biomass, and high protein and oil content in seeds (Kurlovich et al., 1991). The accessions from Algeria were distinguished for their thermal neutrality and high productivity. Altogether, Vavilov enriched the collection with 109 accessions of different Lupinus spp.: 54 from the Mediterranean countries, 9 from both Americas, and 46 from Western Ukraine and Belarus. There were 12 accessions of L. angustifolius among them. During his last expedition to Western Ukraine, Vavilov found an alkaloid-free sample of narrow-leaved lupine; it was added to the collection on November 16, 1940.

In the 1930s and 1940s, the collection continued to be actively expanded at the expense of, inter alia, the material from experiment and breeding stations, e. g., Novozybkov Experiment Station, where the breeding work with lupine began in 1925, and seed samples (for the most part, local varieties and breeding material) from Belarus and Ukraine, obtained by collecting teams during plant explorations.

During the war against the Nazi occupants, some of the preserved germplasm was lost, despite the efforts of VIR’s staff to save the collection. Already in 1945, however, new seed material started to arrive from Novozybkov, Belarusian and Tiraine (Latvia) experiment and breeding stations, and from the Timiryazev Moscow Agricultural Academy.

In the following years, the collection received a lot of breeding material from Belarus and Russia, e. g., from the All-Union Research Institute of Lupine, founded in 1987 (currently a branch of the V.R. Williams Federal Research Center of Forage Production and Agroecology). Germplasm from the German (Gatersleben), Chinese, Australian (CLIMA), Kenyan (National Gene Bank of Kenya, GBK) and other genebanks was constantly added to VIR’s holdings. A significant number of accessions were requested and received by mail from the Polish Lupinus Gene Bank (Poznan, Poland), including cultivars, local populations, and wild forms of L. angustifolius from the centers of origin. Besides, the requested germplasm samples were provided by botanical gardens of the United Kingdom, France, Germany, etc.

In 1991 and 2001, two international collecting teams were sent to Portugal. Thanks to their efforts, the collection was supplemented with 28 accessions, representing local, wild and dedomesticated forms of L. angustifolius. Local varieties were also obtained during an expedition to Brazil.

**Composition of the narrow-leaved lupine collection at VIR**

**Fig. 2.**
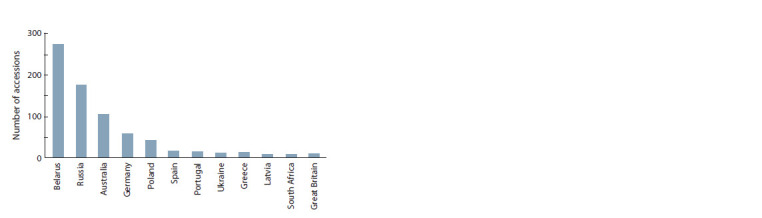
Numbers of L. angustifolius accessions received by VIR from different countries. Only the donor countries of at least 10 accessions are shown.

At present, the collection comprises 887 accessions from 26 countries (Fig. 2).

The largest germplasm shipments came from Belarus, where narrow-leaved lupine breeding dated back to the 1930s. Cv. ‘Rozovy 399’, developed by Y.N. Svirsky, entered the collection in 1945. Cv. ‘Belorussky 155’, bred from a tall white-flowered mutant, was included in the holdings in 1952. Accessions of Belarusian origin are represented by cultivars and breeding material with such valuable traits as earliness, productivity, determinate growth type, nonshattering of pods, absence of alkaloids, disease resistance, etc. Among them, there are many varieties that combine a set of genes significant for breeding, i. e., those determining high protein content, disease resistance, nonshattering of pods, low alkaloid content, etc.

**Fig. 3.**
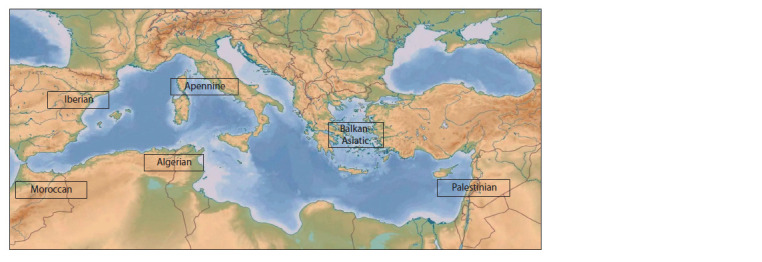
Distribution of L. angustifolius geotypes (ecogeographic groups) across the Mediterranean region – the center of origin for this species (from: Kurlovich et al., 1995).

**Intraspecific classif ications of narrow-leaved lupine used by VIR**

Ecogeographic classification. Researching into the ecotypic structure of the species – a continuation of Vavilov’s doctrine of intraspecific differentiation (Vavilov, 1928, 1962) – resulted in an ecogeographic classification based on the analysis of the VIR collection (Kurlovich et al., 1995). Wild forms are classified into geotypes, or ecogeographic groups of ecotypes. Of the 7 identified geotypes, 6 occur in the Mediterranean region (Fig. 3). Within the geotypes, ecotypes are distinguished: roadside, rocky, mountainous, green manure, etc.

This classification was not widely accepted by researchers and plant breeders. Nevertheless, it reflected the patterns of intraspecific variability within the gene pool, and the knowledge of them can optimize the search for certain genes and traits to be used in the development of specialized cultivars. It was shown that sources for breeding small-seeded cultivars with large biomass should be sought in the Iberian Peninsula. There, in mountainous areas, one can also find sources of cold tolerance as well as plant samples resistant to anthracnose and gray spot. Accessions from the Balkan Peninsula and Palestine can serve as sources for the development of early-ripening and large-seeded cultivars (Kurlovich et al., 1995).

**Agroecological classification.** The cultivated diversity of narrow-leaved lupine is represented by agrogeotypes: Australian, German, Polish, North American, and East European, incorporating different cultivar types (Kurlovich et al., 1995). Cultivar types include cultivar groups of the same utilization trend, with similar biological and agronomic properties.

This classification is based on the history and specificity of plant breeding in different countries, shaped by soil and climate conditions, cultivation techniques, breeding traditions, available source materials, etc. For example, Australian and American cultivars are grown in the autumn/winter season. Therefore, resistance to frosts and returns of cold weather in spring is important for American cultivars. Narrow-leaved lupine cultivars developed in Northern Europe (Gresta et al., 2017), Russia and Belarus should have a short growing season and be adapted to a low sum of mean daily temperatures. Including local varieties and wild accessions from the collection into hybridization makes it possible to transfer frost tolerance or disease resistance to new cultivars (Anokhina et al., 2012). However, European breeders use such material in their work to a limited extent. In the 1960s, lupine breeders in Australia initiated their breeding program based on a number of elite cultivars from Europe and the United States (“founder cultivars”) and observed the effect known as the “bottleneck” of domestication, so they began to actively involve wild forms in crosses. Over time, the proportion of wild ecotypes in the pedigrees of the developed cultivars increased, while the share of “founder cultivars” reduced. As a result of such practice, new high-yielding cultivars were developed in the early 2000s. Their yield exceeded the yield of those released three decades earlier by 81 %, and in addition they acquired resistance to major pathogens and tolerance to herbicides (Cowling, 2020). It should be mentioned that all countries prioritize the development of disease-resistant cultivars.

Agroecological classification indicates specific traits and properties to be looked for in cultivar types as breeding sources. However, due to the progress in plant breeding and substitution of cultivars over the past 25 years, such typification requires upgrading. For example, it ignores cultivar types that serve as sources of traits for breeding cultivars for food or for fish feed in aquaculture, the rapidly developing trends of narrow-leaved lupine utilization.

Botanical classification (Kurlovich, Stankevich, 1990; Kurlovich, 2002; Kuptsov, Takunov, 2006; Vlasova, 2015) systematizes the intraspecific diversity of L. angustifolius according to the color of vegetative and generative organs. The classification identifies varieties of narrow-leaved lupine according to the color of the corolla, jointly with the color and pattern of the seed coat, and its subvarieties according to the color and the presence of anthocyanin on vegetative organs. Determinate and fasciated morphotypes are ranked as forms. Such classification enables crop curators to maintain the authenticity of VIR’s accessions in the process of their reproduction; it is used by breeders for crop testing and by geneticists to establish linkages of genes.

One of the most challenging tasks in morphological characterization of narrow-leaved lupine accessions is to describe the habitus, and features of branching and fruit formation. Difficulties are associated with the absence of a well-established terminology, variability of characters under the effect of the environment, and the presence of transitional forms.

** Morphophysiological classification** according to the growth and branching habit of the stem, proposed by N.S. Kuptsov (Kuptsov, 2001), is currently recognized as the most convenient tool for describing the habitus of narrow-leaved lupine accessions. The degree of branching reduction affects the shaping of morphotypes: wild, quasi-wild, pseudo-wild, corymbose, paniculate, spicate, or palmate. The wild type is characterized by indeterminate stem growth and unlimited branching. In this case, the formation of pods and ripening of seeds do not occur synchronously and, as a rule, are delayed. In other morphotypes, branching is to some extent limited (determined) genetically and blocked by inflorescences. Hybridological methods were used to find out the number of genes and the nature of inheritance of traits in different narrowleaved lupine forms with reduced branching (Adhikari et al., 2001; Oram, 2002; Kuptsov, Takunov, 2006).

The examples of accessions with limited branching from the VIR collection are as follows: spicate-type accessions k-3546, k-3695 (Russia), k-3762 (Germany), k-2955, k-3829, k-3830, k-3832 (Belarus), k-3501, k-3502 (Poland); corymbose-type k-3923 (Belarus); paniculate-type k-3646, k-3641 (Russia); palmate-type k-2979, k-2249 (Russia), etc. Different morphophysiological structure of plants determines their biological properties, such as tolerance to crop densification in a monocrop system, growth rates, flowering and maturation synchrony, stable yield, etc.

**Fig. 4.**
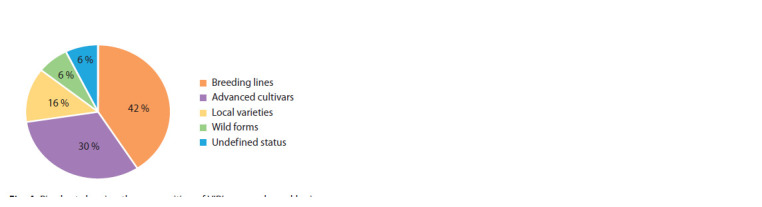
Pie chart showing the composition of VIR’s narrow-leaved lupine collection according to the breeding status of accessions

Breeding status. The collection of L. angustifolius held by VIR includes cultivars developed by scientific breeding (261 accessions), breeding material (370), local varieties (142), wild forms (55), and accessions with an undefined status (50). The percentage of these groups is shown in Fig. 4.

**The results of screening VIR’s narrow-leaved lupine collection for traits of breeding value**

All in all, 640 accessions (73 % of the collection) were assessed for the content of alkaloids in seeds: > 1 % –140 accessions (high alkaloid content); 0.4–1.0 % – 23 accessions; 0.1–0.399 % – 50 accessions; 0.025–0.099 % – 230 accessions (low alkaloid content); and <0.025 % – 197 accessions (alkaloid-free). The data were obtained as a result of a rapid field assessment using Dragendorff ’s reagent (Ermakov et al., 1987) and from published sources. Most of the analyzed accessions (67 %) are low-alkaloid or alkaloid-free. VIR has already tested and selected mass-screening techniques to measure the collection’s alkaloid content through chromatographic methods of analysis, thus making such assessments more accurate (Kushnareva et al., 2020).

The collection was studied for resistance to low temperatures. Sources of cold tolerance were identified (Barashkova et al., 1978).

Biochemical screening for the content of protein and oil in seeds revealed the range of variability of these characters and identified accessions with the highest levels of protein (37.9–39.2 %) and oil (7.5–8.4 %) (Benken et al., 1993).

For quite a long time (1971–1987), the collection was being assessed for resistance to Fusarium under severe infection pressure, including 2–3 infectious environments created artificially by different methods and located in different regions (Bryansk, Kiev, and Leningrad). Accessions with a very high degree of resistance to the disease were identified, including k-2166, k-2167 (Poland), k-1908, k-2266 (Russia), and k-74 (Belarus). Most of narrow-leaved lupine accessions were tested to identify their maturity group and 1000 seed weight (Kiselev et al., 1981, 1988, 1993; Kurlovich et al., 1990).

For many years, VIR’s collection of narrow-leaved lupine has been studied at two sites: Mikhnevo Settlement, Stupino District, not far from Moscow, in the Central Non-Black Earth Region of Russia, and the town of Pushkin near St. Petersburg in the Northwestern Region of Russia. The climate in Stupino District is temperate continental, the mean sum of annual active temperatures for many years is 2000–2200 °C, the precipitation amount is 379 mm, and the growing season lasts 130–135 days. The climate in Pushkin is temperate and humid, transitional from oceanic to continental, the mean sum of annual active temperatures for many years is 1879 °C, the precipitation amount is 637 mm, and the growing season is 105–125 days. It should be mentioned that under the conditions of Pushkin not all narrow-leaved lupine accessions had enough time to develop mature seeds.

Field phenotyping includes the assessment of main agronomic traits: yield, maturity group, susceptibility to diseases, and yield components, such as branching, number of pods per plant, seed weight per plant, and 1000 seed weight.

In 2009– 2019, accessions with limited branching (a gene pool generated from natural and induced mutations) were studied in Stupino. In Pushkin, narrow-leaved lupine accessions of various origin and status were examined for many years. Comparison of the results showed a smaller range of variation in productivity traits in lupine forms with determinate branching, represented by modern cultivars and breeding material from Russia, Belarus, and European countries (Germany, Poland, Latvia, etc.), versus the very heterogeneous material studied in Pushkin. A lower but stable yield, limited plant height, and the number of branches make such mutants suitable for growing in dense stands, when it is easier to control weeds. They do not require defoliation to accelerate their maturation, and, on the whole, their harvestability increases (Table 1).

**Table 1.**
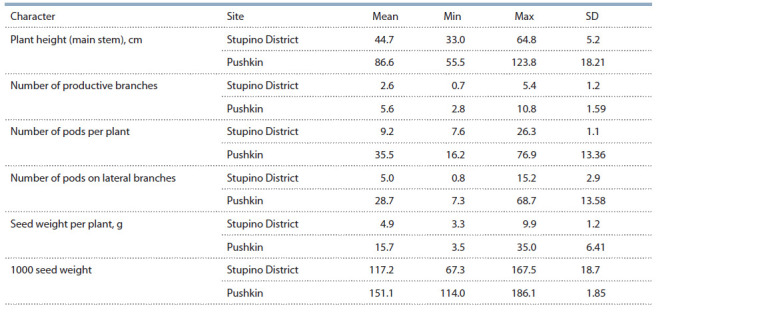
Values of main seed yield characters in narrow-leaved lupine accessions from VIR, assessed in Stupino (near Moscow) and Pushkin (near St. Petersburg)

Comparing traits of breeding value in the accessions with limited branching to those in the varieties with indeterminate growth (wild morphotype) under the conditions of Stupino District testified to a lower seed yield in the former group due to a small number of pods developed on lateral branches. During eight years with hot summers, accessions with different branching patterns had similar growing seasons: 60–80 days. But over the course of four years, with summer temperatures dropping to mean values for many years, indeterminate narrow-leaved lupine forms had difficulties with seed development, unless defoliants were applied, or with afterripening, in contrast to determinate ones. Thus, spicate-type accessions have advantages when cultivated in the northern and northwestern regions of the country due to their earliness and synchronous seed maturation.

For the Central Non-Black Earth Region, the main area of narrow-leaved lupine production, a complex of herbivores afflicting lupine crops was identified. The most harmful pests and diseases were Cerathophorum setosum Kirchn., Thielaviopsis basicola (Berk. & Broome) Ferraris, Fusarium sambucinum Fuckel, Alternaria tenuissimma (Kunze) Wiltshire, Pythium mamillatum Meurs, and Cylindrocladium spp. (Golovin, Vlasova, 2015).

In the Russian Northwest, there were massive infestations of lupine aphids (Macrosiphum albifrons Essig), symptoms of viral diseases caused by Phaseolus virus 2 Smith (BYMV, Bean yellow mosaic virus) and Cucumis virus 1 Smith (CMV, Cucumber mosaic virus). Among the pathogenic fungi, a dominance of representatives of the genera Fusarium Link, Botrytis P. Micheli ex Pers., Sclerotinia Fuckel and Stemphylium Wallr was observed; saprotrophic fungi from the genera Alternaria Nees, Cladosporium Link and Epicoccum Link were also found. Anthracnose incidence, caused by Colletotrichum gloesporioides (Penz.) Penz. & Sacc., was insignificant in both regions.

**Comparative analysis of the world’s narrow-leaved lupine GR collections according to a set of parameters**

Collection size. The world’s largest collection of narrowleaved lupine (2,165 accessions) belongs to Australia (CLIMA). The collection of VIR is the second (887 accessions), followed in decreasing order by the collections of the Scientific and Practical Center of the National Academy of Sciences of Belarus (690), Spain (542), Belarusian State University (371), Poland (361), Portugal (291), Germany (279), and the United States (190). The numbers are given according to the European Central Lupinus DB, the authors of this publication (VIR’s collection), and Privalov et al. (2020) (Belarusian collections).

Status of accessions. Analysis of the breeding status of 3,894 narrow-leaved lupine accessions presented in the European Central Lupinus DB suggests that comparing our collection with the world’s collections according to this criterion is problematic. First of all, not all categories of “the status” are understood in the same way. For example, in the European DB there are very few local varieties (1.8 %), while in the VIR collection 16 % are recognized as such. Secondly, the category “weedy” with reference to narrow-leaved lupine appears only in the databases of the Spanish and Portuguese GR collections of L. angustifolius. There is no such category in the collection of VIR. In some genebanks, the genus Lupinus is represented without any species differentiation. A number of databases do not identify the status of accessions. Mapping populations are present only in the Australian collection, and mutants only in the Australian and Polish collections. That is why any differentiation of the world’s narrow-leaved lupine GR according to the status of their accessions is to a certain extent arbitrary. One should recognize as the absolute fact that wild forms prevail over other narrow-leaved lupine accessions in the world’s GR collections. For example, they account for 82 % in the collection of the PGR Center in Spain and for 60 % in the national collection of Australia (CLIMA).

The results of our analysis demonstrate that on average the global gene pool of narrow-leaved lupine harbors 62 % of wild forms, 12 % of breeding material (lines, hybrids,mapping populations, etc.), 11 % of cultivars developed by scientific breeding, 11 % of accessions with an undefined status, 2 % of mutants, 1.7 % of local varieties, and 0.7 % of weedy accessions. 

The analysis of an older but more representative source that included 5,684 narrow-leaved lupine accessions from 17 collections of the world (Buirchell, Cowling, 1998) also showed the predominance of wild forms and local varieties in those collections. At that time, there were 70 % of them in the Australian collection, 100 % in the collection of Portugal, from 80 to 100 % in three Spanish collections, 78 % in the German collection (Braunschweig), and about 50 % in Poland.

The collection of narrow-leaved lupine at VIR presently consists of the following proportions: 42 % of breeding material, 30 % of cultivars developed by scientific breeding, 16 % of local varieties, 6 % of wild forms, and 6 % of accessions with an undefined status. It means that VIR’s narrow-leaved lupine collection possesses significantly more germplasm partially modified by plant breeding (breeders’ cultivars and breeding material) than all the world’s collections of the species, although the percentage of wild forms is incomparably smaller than in other collections. Meanwhile, the most productive Australian alkaloid-free cultivars were developed by crossing cultivated and wild genotypes, despite the fact that their collection contains elite cultivars from other countries. Australian experts believe that wild forms are also fundamentally important for future improvement of narrow-leaved lupine (Gladstones et al., 1998; Mousavi-Derazmahalleh et al., 2018a; Cowling, 2020).

Phenotypic data. Comparison of these data would be possible only if we refer to a few traits assessed both in the accessions from VIR’s collection and those from the Australian genebank (CLIMA). This comparative analysis shows a greater range of variability in these traits in Australian accessions (Table 2). It can be explained by the predominance of wild forms, recombinant inbred lines, mutant and hybrid populations in the Australian holdings, as their aggregate genetic diversity is much wider than that in VIR’s collection, where cultivars of scientific breeding and breeding material prevail.

**Table 2.**
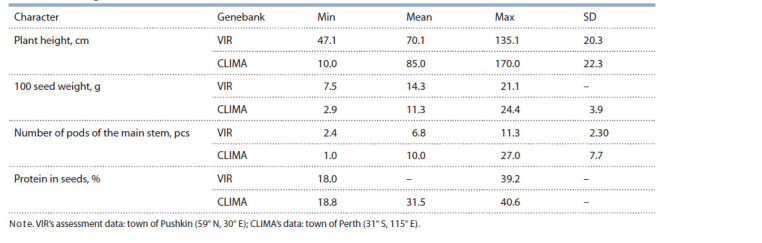
Values of some phenotypic characters in the narrow-leaved lupine collections held by VIR and СLIMA (Buirchell, Cowling, 1998)

**Genetic diversity of wild and cultivated narrow-leaved lupines, identified in different collections**

**Fig. 5.**
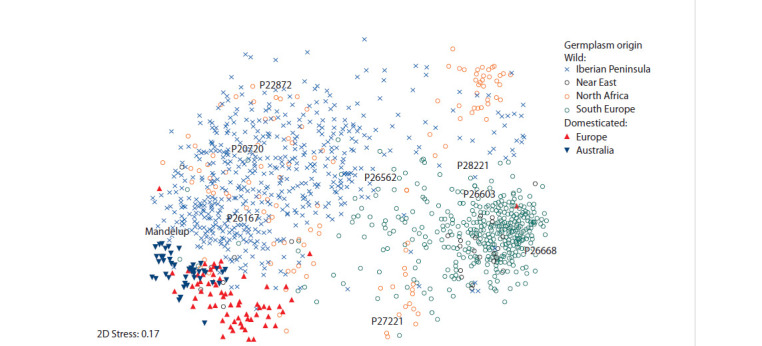
A multidimensional space scatter plot for L. angustifolius accessions from the Australian collection, based on the data from 137 DArT markers (Berger et al., 2012a) classifying accessions according to their domestication status and origin. The designated accessions (P20720, P22872, P26167, P26562, P26603, P26668, P27221, and P28221) were used in enriching BC2 crosses with cv. ‘Mandelup’ (from Berger et al., 2013).

As in most cultivated plants, there is less genetic diversity in domesticated narrow-leaved lupine forms than in wild populations and local varieties, while breeders deal with only a small part of this diversity (Berger et al., 2012a, b). The narrow genetic base of modern cultivars, compared to wild plant forms collected in Southern Portugal, was proved using AFLP and ISSR markers for the analysis of accessions from the Portuguese genebank (Talhinhas et al., 2006). Genotyping the Australian collection of L. angustifolius with 137 DArT (Diversity Array Technology) markers showed clear differences between the wild gene pool, collected in various parts of the Mediterranean region, and modern cultivars. At the same time, a difference was revealed between the cultivars developed in Europe and those from Australia, with the presence of a group of cultivars with “overlapping” traits and properties (Berger et al., 2013) (Fig. 5).

**Methods for identifying differentiation in the gene pool preserved in genebank collections around the world**

Today, molecular genetic methods are the most effective tool to identify the degree of diversity and differentiation in a crop gene pool. The Australian narrow-leaved lupine collection was thoroughly studied. There is detailed information on the habitats of its accessions: latitude, longitude and altitude of the sites where they were collected, together with the data describing local climate and soil conditions, often contrasting in these characteristics. Mass phenotyping of the collection and molecular analysis with DArT markers are being performed. Genetic control of key characters and its molecular mechanisms are being studied (Berger, 2013).

Phenotyping and marker-assisted molecular analysis of accessions from the Portuguese collection of narrow-leaved lupine (Instituto Superior de Agronomia Gene Bank) identified three clearly distinguishable large groups of accessions: 1. Mainly forage accessions (one third of them are breeders’ cultivars, while the remaining part is breeding material from Europe), combining domestication traits (white flowers, large seeds, water-permeable seed coat, and nonshattering pods). However, their plant habitus is close to the wild type: they are tall and prolifically branching. Flowers contain little anthocyanin. 2. Mostly wild forms, plus several cultivars and breeding lines with strongly shattering pods, petals abundant in anthocyanin, and water-impermeable seed coat. Late-flowering genotypes predominate. 3. Mostly cultivars and breeding lines with low weight of the main stem, relatively short branches, very large seeds, and very large nonshattering pods.

AFLP and ISSR marker techniques grouped modern cultivars as subclusters within an extensive variety of wild germplasm, pointing to a narrower genetic base for domesticated forms (Talhinkas et al., 2006).

Polymorphism of the narrow-leaved lupine gene pool in the content of alkaloids in seeds was demonstrated by the researchers who studied 329 accessions from the Polish genebank in Wiatrowo (Kamel et al., 2015). The study included 143 wild forms and populations collected in the sites of their natural occurrence, 108 accessions representing breeding material, and 78 cultivars developed by scientific breeding. The content of alkaloids varied from 0.0005 to 2.8752 % of seed dry weight. Alkaloid content was high in wild forms and low in cultivars, while the accessions of other statuses were predominantly low-alkaloid. In the collection of the Belarusian State University, the content of alkaloids was significantly lower in the seeds of the narrow-leaved lupine cultivars that were developed later, when compared with older cultivars (Sauk et al., 2008). This collection consists of various cultivars developed by domestic and foreign breeders as well as plant forms obtained through mutagenesis and hybridization between cultivars or lines of L. angustifolius.

Almost as soon as plant explorers started collecting samples of wild narrow-leaved lupine forms in the Mediterranean center of the species’ origin, Australian scientists disclosed the crop’s ecogeographic differentiation (Cowling, 1986; Clements, Cowling, 1994), which made it possible to understand the morphophysiological (adaptive) properties of its natural gene pool. It is known that strong relief dissection and dissimilarities of soil and climate conditions have resulted in significant biological and landscape diversity in this vast territory. Seasonal precipitation, temperatures, relative humidity, insolation, and wind speed are highly variable within the Mediterranean basin (Hijmans et al., 2005). The efforts of Australians to procure knowledge of the ecotypic differentiation in the gene pool of narrow-leaved lupine, in their essence, may be recognized as a continuation of the work initiated by Nikolai Vavilov, who was the first to draw attention to the fact that “species occupying significant areas often demonstrate sharply different ecogeographic complexes of forms” (Vavilov, 1965, p. 246). Almost a hundred years after Vavilov (Vavilov, 1928, 1962), Australian scientists arrived to a similar conclusion, confirming that the identification of adaptabilities in a large number of genotypes across different ecological niches makes it possible to find the address of their further production as crops under appropriate conditions (Berger et al., 2017).

Studying the diversity of narrow-leaved lupine ecotypes in the Mediterranean region facilitated our understanding of the species’ reproductive strategy: earliness, and a reduced demand for vernalization and seed dormancy in areas with low precipitation and with droughts in the end of the season. Under such conditions plants bloom earlier, ripen faster, form larger seeds and less biomass, which increases the harvesting index when plant productivity is reduced. The opposite situation is observed in the environments with higher moisture availability and, at the same time, colder weather. Considering these data, phenology can be regarded as a key attribute for the adaptation of wild populations of a species to various habitats within the boundaries of its natural occurrence, and domesticated forms of the species to cultivation areas around the globe (Taylor et al., 2020). The next step is to identify regions associated with climatic adaptation within the genome, in particular, with earliness (Mousavi-Derazmahalleh et al., 2018a).

Genetic variability and phenotypic plasticity were also observed in the architectonics of the root system, when various soil conditions were simulated for growing wild L. angustifolius genotypes (Chen et al., 2011).

Thus, the range of studies, revealing the global genetic diversity of narrow-leaved lupine in order to give it the status of a valuable feed and food crop with good adaptive properties and stable productivity, is quite wide. The prospects for improving the crop, considering its youth, are vast. Key traits that determine its economic importance have been identified. There are tools facilitating the search for the sources of these traits in the worldwide gene pool of the species. It is necessary to combine the efforts of scientists, collection holders and plant breeders to exchange the crop’s genetic resources in order to enhance its genetic diversity. This need has been tirelessly voiced by Australian experts, working with the GR of narrow-leaved lupine (Buirchell, Cowling, 1998; Berger, 2013; Cowling, 2020; etc.).

## Conclusion

The collection of narrow-leaved lupine GR preserved at VIR is represented by a wide variety of accessions with different statuses. Cultivars developed by scientific breeding and breeding material prevail among them. A special place in this gene pool is occupied by accessions with limited branching, most adapted to cultivation in relatively northern regions. They are early-ripening, demonstrate lower but more stable productivity, and are suitable for cultivation in densified stands, which offers a number of agronomic advantages. Comparison of VIR’s collection of L. angustifolius germplasm with other national collections in the genebanks of lupine-producing countries shows that there are very few wild forms in it. Meanwhile, the collections of other genebanks are rich in wild genotypes. Australia, where the crop’s wild gene pool was actively involved in breeding programs, has impressively succeeded in the use of narrow-leaved lupine GR for the development of high-yielding cultivars.

Specific features of the species’ reproductive strategy have so far been established, so it can now be adapted to a wide range of environmental conditions. All this calls for further disclosure and exploitation of narrow-leaved lupine’s genetic and ecotypic potential, including its wild forms and local varieties, to promote more intensive breeding efforts and large-scale production of this crop in Russia for feed and food purposes. Introgression of the adaptability traits, found in wild and locally cultivated plant forms, into modern cultivars will help to expand the production area of narrow-leaved lupine. This task requires enhancing breeding, genetic, physiological, biochemical and metabolomic studies of the crop’s gene pool as well as developing its genomic resources. Identification of the genome regions associated, inter alia, with earliness will immeasurably increase the efficiency of searches for source material promising for Russian breeding in GR collections.

## Conflict of interest

The authors declare no conflict of interest.
